# Integrative single-cell and cell-free plasma RNA transcriptomics identifies biomarkers for early non-invasive AD screening

**DOI:** 10.3389/fnagi.2025.1571783

**Published:** 2025-05-30

**Authors:** Li Wu, Renxin Zhang, Yichao Wang, Shaoxing Dai, Naixue Yang

**Affiliations:** ^1^State Key Laboratory of Primate Biomedical Research, Institute of Primate Translational Medicine, Kunming University of Science and Technology, Kunming, Yunnan, China; ^2^Yunnan Key Laboratory of Primate Biomedical Research, Kunming, Yunnan, China

**Keywords:** Alzheimer’s disease, cell-free RNA, single-cell RNA-seq, machine learning, non-invasive early screening

## Abstract

**Introduction:**

Data-driven omics approaches have rapidly advanced our understanding of the molecular heterogeneity of Alzheimer’s disease (AD). However, limited by the unavailability of brain tissue, there is an urgent need for a non-invasive tool to detect alterations in the AD brain. Cell-free RNA (cfRNA), which crosses the blood-brain barrier, could reflect AD brain pathology and serve as a diagnostic biomarker.

**Methods:**

Here, we integrated plasma-derived cfRNA-seq data from 337 samples (172 AD patients and 165 age-matched controls) with brain-derived single cell RNA-seq (scRNA-seq) data from 88 samples (46 AD patients and 42 controls) to explore the potential of cfRNA profiling for AD diagnosis. A systematic comparative analysis of cfRNA and brain scRNA-seq datasets was conducted to identify dysregulated genes linked to AD pathology. Machine learning models—including support vector machine, random forest, and logistic regression—were trained using cfRNA expression patterns of the identified gene set to predict AD diagnosis and classify disease progression stages. Model performance was rigorously evaluated using area under the receiver operating characteristic curve (AUC), with robustness assessed through cross-validation and independent validation cohorts.

**Results:**

Notably, we identified 34 dysregulated genes with consistent expression changes in both cfRNA and scRNA-seq. Machine learning models based on the cfRNA expression patterns of these 34 genes can accurately predict AD patients (the highest AUC = 89%) and effectively distinguish patients at early stage of AD. Furthermore, classifiers developed based on the expression of 34 genes in brain transcriptome data demonstrated robust predictive performance for assessing the risk of AD in the population (the highest AUC = 94%).

**Discussion:**

This multi-omics approach overcomes limitations of invasive brain biomarkers and noisy blood-based signatures. The 34-gene panel provides non-invasive molecular insights into AD pathogenesis and early screening. While cfRNA stability challenges clinical translation, our framework highlights the potential for precision diagnostics and personalized therapeutic monitoring in AD.

## Introduction

As the most prevalent cause of dementia, Alzheimer’s disease (AD) is a progressive neurodegenerative disorder characterized by memory loss, cognitive decline and behavioral impairments. The pathology of AD included synaptic loss (DeKosky and Scheff, 1990; Masliah et al., 1991; Terry et al., 1991), neuroinflammation (Leng and Edison, 2021), oxidative stress (Tönnies and Trushina, 2017), misfolded proteins (de Calignon et al., 2012; Moreno-Gonzalez and Soto, 2011), and mitochondrial dysfunction (Swerdlow et al., 2014), culminating in neuronal death and brain dysfunction (Horwich, 2002). Currently, no effective treatment strategies have been established to prevent or slow down the progression of AD (Cummings et al., 2023; Peng et al., 2023). Given the absence of effective diagnostic methods for the aforementioned etiologies in clinical practice, resulting in the majority of AD patients being diagnosed at advanced stages of the condition. However, traditional diagnostic methods, such as neuroimaging and cerebrospinal fluid (CSF) analysis, can be invasive and costly (Boerwinkle et al., 2021; Henjum et al., 2022; Karikari et al., 2021; Shaw et al., 2009). CSF analysis for AD diagnosis primarily relies on measuring biomarkers, including amyloid-beta (Aβ), total tau (t-Tau), and phosphorylated tau (p-Tau) (Olsson et al., 2016; Paterson et al., 2018; Shaw et al., 2009). Studies suggest that CSF biomarkers can serve as an early diagnostic tool for AD before cognitive impairment becomes apparent, by detecting Aβ42 and p-Tau in the CSF, AD can be identified at an earlier stage (de Leon et al., 2004; Papaliagkas et al., 2023). However, the methods, reagents, and reference ranges for CSF biomarker testing are not yet standardized, leading to variations in diagnostic performance (Hansson et al., 2018; Molinuevo et al., 2014). In parallel, a variety of precise and robust analytical techniques, including mass spectrometry and automated ultrasensitive immunoassays, have been established for quantifying plasma concentrations of AD-related biomarkers (Cai et al., 2023; Stockmann et al., 2020; Teunissen et al., 2022), such as Aβ (West et al., 2021), t-Tau, p-Tau (Karikari et al., 2020; Mielke et al., 2018; O’Connor et al., 2021; Palmqvist et al., 2020; Thijssen et al., 2020; Thijssen et al., 2021), neurofilament light chain (NfL) (Olsson et al., 2016). Numerous studies have demonstrated its clinical value and accuracy in detecting pathological changes in AD by measuring plasma amyloid-β_42:40_ ratio and the levels of p-Tau 181 and p-Tau 217 in clinically defined patients (Mielke and Fowler, 2024). However, the correlation between plasma biomarkers and CSF biomarkers still needs further research to ensure their accuracy (Ashton et al., 2023; Wojdała et al., 2023).

A potential breakthrough in this area may come from the recent advancements in blood-based biomarkers (BBMs), which could provide a valuable resource for studying molecular changes with non-invasive procedures, avoiding traditional surgical risks and discomfort. BBMs allow for sequential sampling, enabling the monitoring of disease progression and prediction of pharmacological responses. In cancer detection, BBMs broadly include circulating tumor cells (CTCs) (Haber and Velculescu, 2014; Habli et al., 2020; Lin et al., 2021), circulating tumor DNA (ctDNA) (Haber and Velculescu, 2014; Li and Sun, 2024), tumor-derived exosome and cell free DNA or RNA (Larson et al., 2021), which facilitate real-time tracking of tumor dynamics, addressing heterogeneity and supporting the development of personalized treatment strategies (Ho et al., 2024; Onidani et al., 2019; Passaro et al., 2024). However, the presence of the blood-brain barrier poses a significant challenge in developing BBMs for neurological diseases, slowing progress in this area compared to cancer research. The development of BBMs for neurodegenerative disorders like AD remains more complex and challenging. A few studies suggest that blood-derived Aβ and tau serve as cost-effective alternative to traditional CSF-based markers for AD diagnosis. Evidence indicates that blood Aβ42/Aβ40 ratios may reflect Aβ pathology earlier than CSF markers (Cai et al., 2024). In addition, the levels of miRNAs from peripheral blood can accurately predict p-Tau/Aβ42 ratio in CSF, indicating potential for a non-invasive protocol for early screening and diagnosis of AD (Campbell et al., 2021; Jia et al., 2021; Wang J. et al., 2018). However, measuring levels such as Aβ and Tau protein is not sensitive and straightforward enough for accurate diagnosis of incipient AD. There is an urgent need for novel BBMs that originate directly from brain lesions and accurately reflect the underlying mechanisms of the lesions.

Cell-free RNA (cfRNA), known as extracellular RNA, consists of RNA fragments originating from both healthy and diseased cells across various tissues and can be found circulating freely in the blood. CfRNA detected in blood offers a non-invasive method to directly assess the status of multiple tissues. Current applications of cfRNA span various fields such as cancer detection (Larson et al., 2021; Roskams-Hieter et al., 2022; Tao et al., 2023), bone marrow transplantation (Loy et al., 2024; Toden et al., 2020), obstetrics (Moufarrej et al., 2022; Rasmussen et al., 2022), neurodegeneration (Wen et al., 2021), tuberculosis (Chang et al., 2024), and liver disease (Chen et al., 2017; Mann et al., 2018; Zhou et al., 2019). CfRNA markers effectively track cancer progression, predict patient survival outcomes, and reveal tissue- and subtype-specific biomarkers for cancer (Larson et al., 2021). Certainly, cfRNA also reflect brain characteristics of neurological disorders. A recent investigation using blood messenger cfRNA identified genes associated with dementia severity. These genes are significantly enriched in biological processes linked to AD pathology, such as abnormal synaptic function, mitochondrial dysfunction, and inflammatory responses (Toden et al., 2020). Importantly, cfRNA has been demonstrated to accurately distinguish AD patients from healthy control, with dysregulated genes clustering in patterns closely associated with AD progression. It has been reported that blood cf-miRNAs biomarkers associated with AD are concordance with neuropsychological and neuroimaging assessments (Cheng et al., 2015). Furthermore, research has confirmed that the expression levels of cf-miRNAs in the blood can distinguish between cognitively normal individuals and patients with mild cognitive impairment as well as AD patients (Sheinerman and Umansky, 2013; Sheinerman et al., 2012). Such growing evidence supports the notion that RNA molecules can traverse the blood-brain barrier, with brain-derived cfRNAs detected in blood serving as promising biomarkers for non-invasive molecular profiling of neurological disorders such as AD.

There is a need for a more detailed understanding of physiological origins of cfRNA at the cellular resolution. The advent of single-cell technology offers a high-resolution approach to investigate the brain pathology in AD patients. Recent studies utilized single-cell transcriptome techniques to reveal the molecular changes in excitatory neurons and oligodendrocytes in AD (Mathys et al., 2023). Transcriptional changes in astrocytes and microglia in AD involve upregulation of neuroprotective genes that regulate cell homeostasis, phagocytosis, and clearance of Aβ and p-Tau (Smith et al., 2022). Analysis of a rare cortical biopsy cohort also revealed significant enrichment of early cortical amyloid responses in neurons, as well as heightened neuroinflammatory responses in microglia and upregulated β-amyloid gene expression in oligodendrocytes (Gazestani et al., 2023). Notably, the APOE4 gene variant has been found to increase the risk of AD by disrupting cholesterol homeostasis in oligodendrocytes and affecting myelin formation (Blanchard et al., 2022), and APOE4 carriers may exhibit an accelerated breakdown of the blood-brain barrier before the onset of cognitive impairment (Montagne et al., 2020). Despite significant advances in single-cell technology in AD studies, there is currently no literature directly linking cfRNA to biopsies of AD for non-invasive monitoring of disease progression and therapeutic efficacy.

In this study, we collected blood-derived cfRNA sequencing data from a cohort of 337 samples, comprising 172 AD samples and 165 age-matched control (Toden et al., 2020). Total of 431 up-regulated and 2,658 down-regulated genes were identified in AD patients. These dysregulated genes are enriched in proinflammatory biological processes and impaired nervous system function, suggest that blood cfRNA may detect the pathological features characteristic of AD. However, the classifier models based on cfRNA dysregulated genes lack the robustness and accuracy to distinguish AD patients effectively. To explore the potential of cfRNA profiling in detecting AD brain lesions, we integrated cfRNA-seq data with brain-derived single cell RNA-seq (scRNA-seq) data from 88 samples. Systematic profiling of cfRNA has revealed its potential to non-invasively detect alterations in cell-type-specific signatures within the AD brain, as inferred from scRNA-seq analysis. Finally, we identified a total of 34 key biomarker genes that exhibited the highest importance scores in our feature selection algorithm and were differentially expressed in both cfRNA and scRNA datasets. Machine learning models based on the cfRNA expression patterns of these 34 genes can accurately predict AD patients (the highest AUC=̃ 89%). Moreover, classifiers developed based on the expression of 34 genes in brain transcriptome data also demonstrated robust predictive performance for assessing the risk of AD in the population (the highest AUC = 94%). These models were capable of effectively identifying patients in the early stages of AD, which is critical for initiating timely therapeutic interventions. These findings highlight the potential of these 34 genes as biomarkers for early non-invasive screening of AD, paving the way for enhanced diagnostic accuracy and patient stratification in AD research and clinical practice.

## Materials and methods

### cfRNA data preprocessing

We downloaded the cfRNA raw Sequence Read Archive (SRA) data from public repositories (PRJNA574438) and utilized the fastq-dump (version 3.0.2) pipeline to convert it into FASTQ files. The data then was processed by quality control using fastp (Chen et al., 2018) (version 0.23.2), followed by gene expression counting with featureCounts (Liao et al., 2014) (version 2.0.3). Based on the metadata information, 172 AD patients and 165 age-matched control were obtained (Supplementary Table 1).

### Identification of differentially expressed genes (DEGs) of cfRNA data

Subsequent differential expression analysis was conducted using DESeq2 (Love et al., 2014) Bioconductor package (version 1.44.0). Briefly, raw count data were imported into a DESeqDataSet object via the *DESeqDataSetFromMatrix* function. To account for technical variability, the analysis incorporated batch correction based on sample collection sources (multiple medical centers), while normalizing for sequencing depth. Variance-stabilizing transformation (VST) was applied using the *vst* function, and normalized expression values were extracted via the *assay* method. Differential expression testing between AD patients and control was performed using the default Wald test within the DESeq workflow, with results extracted via the *results* function. To address multiple hypothesis testing, we applied the Benjamini-Hochberg procedure to control the false discovery rate (FDR) at a threshold of 0.05. This stringent filtering identified 2,658 significantly downregulated genes [adjusted *p*-value (*p*_*adj*_) ≤ 0.05 and log2 fold change (log2FC) < 0] and 431 up-regulated genes (*p*_*adj*_ ≤ 0.05 and log2FC > 0) in AD patients relative to age-matched control (Supplementary Table 2).

### Integrating and quality controlling single-cell RNA data

We downloaded the scRNA-seq data from 88 brain samples, comprising 46 AD patients and 42 age-matched control. These samples originated from four distinct regions (Supplementary Table 3): hippocampus (HIP: GSE185553, GSE185277, GSE198323, GSE163577), frontal prefrontal cortex (FPC: GSE163577, GSE157827, GSE174367), frontal cortex (FC: GSE222494, GSE163577) and entorhinal cortex (EC: GSE138852) and processed the data using both R and Python environments. Scanpy (Wolf et al., 2018) package (version 1.9.3) was used to perform processing according to the standard pipeline: Cells with less than 200 Unique Molecular Identifier (UMI) counts, over than 7,500 genes, or over seven mitochondrial RNA counts were filtered out, and genes expressed in less than three cells were removed as well. The filtered expression matrix was normalized and log2-transformed. Finally, the filtered matrix contains 603,636 cells, 1,287 genes and 2,704 counts per cell, on average.

### Batch effect correction

Single cell RNA sequencing (scRNA-seq) and single nuclei RNA sequencing (snRNA-seq) datasets derived from multiple independent studies were integrated into a unified expression matrix using the *sc.concatenate* function in Scanpy. Highly variable genes (HVGs) were identified through variance stabilization of the normalized data using Scanpy’s *sc.pp.highly_variable_genes*. Principal component analysis (PCA) was subsequently applied to the HVGs’ expression matrices with *sc.tl.pca*. A k-nearest neighbor graph was constructed using *sc.pp.neighbors*, followed by Uniform Manifold Approximation and Projection (UMAP) visualization with *sc.tl.umap* to reveal cellular clusters in two-dimensional space, all with default parameters. Then, we systematically assessed potential batch effects across biological covariates: data source (different published studies), sequencing platform (scRNA-seq vs. snRNA-seq), diagnostic groups (AD vs. control), and neuroanatomical regions (four distinct areas).

In our dataset, before batch correction, cells primarily clustered by data source samples failing to integrate effectively. To address batch effects arising from heterogeneous data sources, we implemented batch effect correction for each data source using the bbknn (Polański et al., 2019) package (v1.6.0) through *sc.external.pp.bbknn (adata, batch_key* = “*sources*”). Subsequently, we applied UMAP analysis with 30 nearest neighbors via *sc.tl.umap* to visualize the bbknn-harmonized data and identify cell clusters within the UMAP embedding space. Additionally, using classical markers, we performed cell annotation. Finally, to facilitate visualization, we transformed the data into the Seurat (Butler et al., 2018) (version 4.3.0) format and utilized ggplot2 (version 3.4.2^1^) for data visualization and esthetic refinement.

### Pseudo-bulk analysis of single-cell RNA

Due to the intrinsic sparsity of single-cell sequencing data, we have utilized the pseudo-bulk method to standardize gene abundance levels in both the single-cell data and cfRNA data. In detail, for each cell type, we employed a non-replacement random sampling method separately between the AD and the normal aging groups, selecting 4,000 cells to constitute the first pseudo-sample. Subsequent sampling was performed to form additional pseudo-samples, and this process was repeated until all cells had been fully sampled. The final sample for each group included any cells that had not been included in the previous rounds. Thereafter, differential expression analysis was conducted between the AD and control groups using the DESeq2 package, with genes filtered for further analysis if they had a *p*_*adj*_ ≤ 0.05 and |log2FC| ≥ 0.25. The pseudo-bulk results can be found in Supplementary Table 4.

### Enrichment analysis

To assess the distinct biological functions of AD in contrast to the normal aging process, the R package clusterProfiler (Wu et al., 2021) (version 4.1.1) was employed, using its enrichR function for conducting Kyoto Encyclopedia of Genes and Genomes (KEGG) and Gene Ontology (GO) pathway enrichment analyses. The criteria for selecting significant pathways for visualization were *p*_*adj*_ ≤ 0.05 and count ≥ 3. Additionally, any KEGG pathways associated with “cancer” were excluded from this analysis.

### Quantification of group enrichment analysis

To determine the group-specific enrichment of distinct cell types within the single-cell landscape of AD and normal aging, we performed statistical analyses using the R_*o/e*_ (Ratio of Observed to Expected) approach for various cell types, following the protocol described by Zhang et al. (2018). For any specific cell cluster, an R_*o/e*_ value greater than 1 indicates a significant enrichment within a particular group, while a value less than 1 indicates a notable depletion within that group.

### Overview of AD diagnostic classifier model training

To optimize classifier performance evaluation and reduce potential bias and overfitting, we utilized AD and normal aging control from UCSD and BioIVT as the validation cohort, while samples from all other sources served as the training cohort. It is important to note that samples within the validation cohort were not utilized in any form during the model training process. After feature selection process (the following description), we applied the sklearn Recursive Feature Elimination with Cross-Validation (RFECV) algorithm to meticulously select 47 genes from the cfRNA data and 34 genes (Supplementary Table 5) from the merged cfRNA and scRNA datasets. These genes were then, respectively input into the downstream classifier algorithms for further analysis. The expression levels (standardized counts data) of those genes were then used in the subsequent training of the classifiers using the Python (version 3.7.16) library scikit-learn.^2^ We have applied three distinct classification algorithms—Support Vector Machine (SVM), Random Forest (RF), and Logistic Regression (LR)—to carry out our classification tasks. Each algorithm was subjected to a 10-fold cross-validation on both the training and validation cohorts. As a result, we were able to generate Receiver Operating Characteristic (ROC) curves and determine the corresponding Area Under the Curve (AUC) values for each classification model.

### Training-validation splitting of multi-source cfRNA-seq cohorts

The cfRNA-seq datasets comprised AD and normal aging control samples collected across multiple medical centers, with the following sample distribution: BioIVT - 0 AD, 30 control; GEMs (Indiana) - 2 AD, 45 control; UCSD - 80 AD, 0 control; University of Kentucky - 90 AD, 41 control; Washington University in St. Louis - 0 AD, 49 control. To ensure robust model evaluation, we stratified the data into independent validation and training/test cohorts: 40 AD samples (randomly subsampled from UCSD, correction: samples previously misattributed to GEMs were in fact from UCSD) and 30 control samples (from BioIVT) were allocated as the validation set, while the remaining samples (UCSD: 40 AD; GEMs: 2 AD, 45 control; University of Kentucky: 90 AD, 41 control; Washington University: 49 control) were used for model training and testing. For detailed cohort composition and stratification criteria, refer to Supplementary Table 1.

### Feature selection and model training in cfRNA-based genes (47 genes)

Initial analysis of cfRNA-seq data applied Benjamini-Hochberg FDR correction (*p*_*adj*_ ≤ 0.05), identifying 2,658 down-regulated and 431 up-regulated genes. To identify high-confidence biomarker candidates, a stricter filter log2FC ≥ 1 and *p*_*adj*_ ≤ 0.05, for up-regulated (38 genes); log2FC ≤ −3 and *p*_*adj*_ ≤ 0.05, for down-regulated (69 genes) yielded 107 high-confidence biomarker candidates. For predictive modeling, we employed Python’s scikit-learn package using RFECV to optimize feature selection. This algorithm iteratively removes the least important features based on classifier weights while monitoring cross-validation accuracy. Through RFECV-optimized feature selection (10-fold cross-validation with random forest classifier), we identified 47 key genes can achieve best predictive accuracy. These key genes demonstrated good classification performance across three models in independent validation cohorts (RF: test AUC = 0.87/valid AUC = 0.66; SVM: test AUC = 0.84/valid AUC = 0.61; LR: test AUC = 0.80/valid AUC = 0.59).

### Feature selection and model training in genes from integrated cfRNA and scRNA datasets (34 genes)

Pseudo-bulk analysis of scRNA datasets revealed 15,209 up-regulated and 18,096 down-regulated genes under Benjamini-Hochberg FDR-adjusted criteria (*p*_*adj*_ ≤ 0.05, |log2FC| ≥ 0.25). Integration of DEGs revealed 112 co-upregulated genes conserved across both scRNA and cfRNA, which were prioritized for downstream biomarker discovery. In feature selection, RFECV ultimately identifying 34 optimal biomarkers, these biomarkers demonstrated best classification performance across three models in independent validation cohorts (RF: test AUC = 0.89/valid AUC = 0.82; SVM: test AUC = 0.81/valid AUC = 0.78; LR: test AUC = 0.80/valid AUC = 0.84).

### The progress of brain-derived bulk RNA-seq data cohorts

The brain-derived bulk RNA-seq data from both AD and normal aging samples, were used to evaluate the performance of our cfRNA-based module in AD assessment: Religious Orders Study and Memory and Aging Project (ROSMAP^3^) (Mostafavi et al., 2018), Mayo Clinic Alzheimer’s Disease Genetics Studies (Mayo^4^) (Bennett et al., 2018), and Mount Sinai Brain Bank (MSBB) (Wang M. et al., 2018). These three independent datasets underwent systematic data harmonization and annotation through the following procedures:

### Data integration and classification for ROSMAP cohorts

The bulk RNA-seq expression matrix (ROSMAP_RNAseq_FPKM_gene.tsv) was integrated with metadata from two sources: ROSMAP_biospecimen_metadata.csv: Specimen-level technical annotations (e.g., brain region dissection, postmortem interval, RNA extraction protocols). ROSMAP_clinical.csv: Longitudinal clinical and neuropathological data (e.g., antemortem cognitive assessments, CERAD neuritic plaque scores, Braak staging). Samples were classified into AD, Mild Cognitive Impairment (MCI), or control groups based on standardized dementia severity criteria outlined in the ROSMAP clinical-pathological protocol. Following quality control, the final cohort comprised 254 neuropathologically confirmed AD cases and 200 cognitively normal control from postmortem brain tissues, which were subsequently used for model training.

### Data integration and classification for Mayo cohorts

Raw bulk RNA-seq expression matrices from two Mayo cohorts:

MayoRNAseq_RNAseq_CER_geneCounts-278.tsv (cerebellar cortex),

MayoRNAseq_RNAseq_TCX_geneCounts-278.tsv (temporal cortex).

These datasets were merged into a unified expression matrix and annotated with metadata from two sources: MayoRNAseq_biospecimen_metadata.csv: Specimen-level technical details (e.g., brain region dissection protocols, RNA integrity numbers). MayoRNAseq_individual_metadata_031422.csv: Donor-level clinical and neuropathological variables (e.g., Braak staging, Thal amyloid phase, APOE genotype). Sample classification into AD or control groups was determined by board-certified neuropathologists based on postmortem histopathological evaluations (e.g., amyloid-β plaques, neurofibrillary tangles). Following rigorous quality filtering, the final cohort comprised 166 neuropathologically confirmed AD cases and 156 control from postmortem brain tissues, which were subsequently used for machine learning model training.

### Data integration and classification for MSBB cohorts

Raw bulk RNA-seq expression matrices from four MSBB cohorts:

AMP-AD_MSBB_MSSM_BM_10.raw_counts.tsv,

AMP-AD_MSBB_MSSM_BM_22.raw_counts.tsv,

AMP-AD_MSBB_MSSM_BM_36.raw_counts.tsv,

AMP-AD_MSBB_MSSM_BM_44.raw_counts.tsv.

The four MSBB cohorts were merged into a unified expression dataset and annotated with metadata from two sources: MSBB_biospecimen_metadata.csv (specimen-level technical details, e.g., brain region, RNA quality metrics) and MSBB_individual_metadata.csv (donor-level clinical and demographic variables, e.g., age, sex, neuropathological diagnoses). Based on dementia severity scores defined in the MSBB clinical protocol, samples were classified into AD, MCI, or control groups. Following rigorous quality filtering, the final cohort comprised 346 postmortem AD cases and 242 neuropathologically confirmed control, which were subsequently used for machine learning model training.

### Model training on 34 genes in brain-derived bulk RNA-seq cohort

Following data preprocessing, we obtained three brain-derived bulk RNA-seq cohorts (ROSMAP: 254 AD vs. 200 control; Mayo: 166 AD vs. 156 control; MSBB: 346 AD vs. 242 control), where 30% of samples from both AD and control groups were allocated as validation sets and 70% as training sets. Using 34 biomarkers co-identified through cfRNA-scRNA datasets integration, model training achieved strong performance. In the training sets: Mayo (RF: AUC = 0.82, SVM: AUC = 0.94, LR: AUC = 0.94), MSBB (RF: AUC = 0.75, SVM: AUC = 0.67, LR: AUC = 0.66), ROSMAP (RF: AUC = 0.69, SVM: AUC = 0.73, LR: AUC = 0.72); and in independent validation sets: Mayo (RF: AUC = 0.86, SVM: AUC = 0.89, LR: AUC = 0.88), MSBB (RF: AUC = 0.69, SVM: AUC = 0.70, LR: AUC = 0.68), ROSMAP (RF: AUC = 0.62, SVM: AUC = 0.63, LR: AUC = 0.58).

## Results

### Blood cell-free RNA facilitates the non-invasive detection of pathological features of AD

**FIGURE 1:**
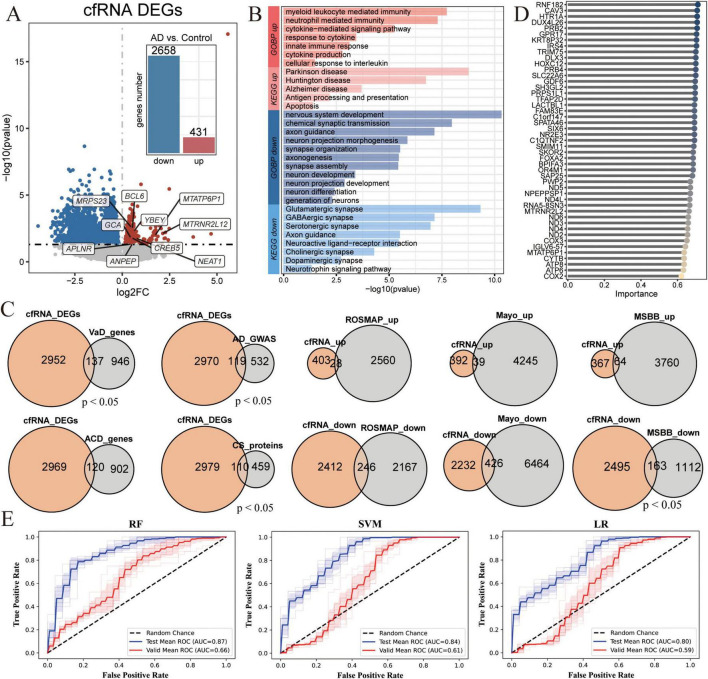
Transcriptomics analysis of cell-free RNA (cfRNA) reveals regulation of Alzheimer’s disease (AD)-associated gene expression changes. **(A)** Volcano plot shows differentially expressed genes (DEGs) in cfRNA-seq between AD (*n* = 172) and age-matched control (*n* = 165). *P*_*adj*_ ≤ 0.05 was used as the cutoff criteria. The bar chart represents the number of up- regulated (red) and down-regulated (blue) genes. Genes with *p*_*adj*_ ≤ 0.05 (Wilcoxon rank-sum) were defined as DEGs. **(B)** Barplot shows the representative significantly (*p*_*adj*_ ≤ 0.05 and count ≥ 3) enriched Gene Ontology (GO) and Kyoto Encyclopedia of Genes and Genomes (KEGG) terms associated with up- and down-regulated DEGs in the cfRNA dataset. **(C)** Venn diagrams illustrate the overlap of DEGs of cfRNA with genes related to Vascular Dementia (VaD) (Guo et al., 2024; Mega Vascular Cognitive Impairment and Dementia (Megavcid) consortium, 2024), All-Cause Dementia (ACD) (Guo et al., 2024; Mega Vascular Cognitive Impairment and Dementia (Megavcid) consortium, 2024), genome-wide association studies (GWAS) geneset for AD (Hahn et al., 2023), and cognitive function -related proteins (CS proteins) (Wingo et al., 2019), brain-derived DEGs in AD from the Religious Orders Study and Memory and Aging Project (ROSMAP) (Mostafavi et al., 2018), Mayo (Bennett et al., 2018), or Mount Sinai Brain Bank (MSBB) geneset (Wang M. et al., 2018). **(D)** Lollipop plot shows the top 47 representative genes, ranked by their importance based on importance in feature selection algorithms. **(E)** The Receiver Operating Characteristic (ROC) curves for the diagnosis of AD patients in the test (blue) and validation (red) set across three models.

Previous blood-derived transcriptome studies have demonstrated the potential to determine the tissue origin of diseases using cfRNA (Vorperian et al., 2022). Despite the presence of a blood-brain barrier between the brain and peripheral blood, astrocyte-specific changes in AD pathology are non-invasively measurable from blood-derived cfRNA (Liddelow et al., 2017; Vorperian et al., 2022). To establish AD-specific cfRNA signatures that could serve as non-invasive biomarkers for the diagnosis of AD, we collected the sequencing data of blood cf-mRNA from 126 AD patients and 116 age-matched healthy control (Toden et al., 2020; Supplementary Figure 1A). We identified 431 up-regulated and 2,658 down-regulated genes in AD patients (Figure 1A). The up-regulated genes were enriched in pathways linked to Parkinson’s disease, Alzheimer’s disease, antigen processing and presentation, and cytokine production. This enrichment result aligns with the pathological perspective that AD patients exhibit a proinflammatory state and are prone to disease progression (Figure 1B). The down-regulated genes showed significant enrichment in pathways crucial for nervous system development, including synaptic organization, axonogenesis, synaptic assembly, neuronal development, and glutamate synapses (Figure 1B). Dysregulation of these pathways, which are essential for normal nervous system function, reflects a loss of functional mechanisms in degenerative diseases. These findings suggest that blood cfRNA may detect the pathological features characteristic of AD.

When we compared the differential expression patterns of cfRNA to the reported gene sets, we found that cfRNA in blood does indeed reflect the characteristics of neurodegenerative diseases. Specifically, there was a significant overlap with gene sets associated with Vascular Dementia (VaD) (Guo et al., 2024; Mega Vascular Cognitive Impairment and Dementia (Megavcid) consortium, 2024), All-Cause Dementia (ACD) (Guo et al., 2024; Mega Vascular Cognitive Impairment and Dementia (Megavcid) consortium, 2024), genome-wide association studies (GWAS) geneset for AD (Hahn et al., 2023), and cognitive function proteins (Wingo et al., 2019; Figure 1C). To further validate this finding, we also conducted association analyses comparing differential expression patterns in AD patients with those in health aging control, utilizing brain-derived RNA-seq data from three databases: Religious Orders Study and Memory and Aging Project (ROSMAP^3^) (Mostafavi et al., 2018), Mayo Clinic Alzheimer’s Disease Genetics Studies (Mayo^4^) (Bennett et al., 2018), and Mount Sinai Brain Bank (MSBB) (Wang M. et al., 2018; Figure 1C). The analysis revealed that a significant number of detected genes exhibiting differential expression in cfRNA were also found to be altered in AD brain lesions. This suggests that cfRNA could be derived from the brain, capable of crossing the blood-brain barrier into the bloodstream, thereby providing a theoretical foundation for the use of cfRNA in non-invasive diagnostic approaches.

To investigate the potential of cfRNA as diagnostic biomarkers for AD, we have deployed a comprehensive machine learning model to evaluate its predictive performance. Specifically, the data were split into training (70%), testing (20%), and validation (10%) sets from the cfRNA cohort with 126 AD patients and 116 age-matched healthy control (Supplementary Figures 1A, D). Three different classifiers were trained including Support Vector Machine (SVM), Random Forest (RF), and Logistic Regression (LR). Utilizing a feature selection RFECV algorithm, we identified 47 key genes for the classification task (Figure 1D and Supplementary Figure 1E). Relatively good classification performance was achieved in the three models, with an AUC ≥ 0.8 (RF: AUC = 0.87, SVM: AUC = 0.84, LR: AUC = 0.80) (Figure 1E). However, in the independent validation cohort, the performance of all three models was suboptimal, with the RF model achieving the highest AUC of only 66%, indicating that cfRNA-based biomarkers may contribute to a higher false positive rate in AD diagnosis. We suspect that the reason for the poor prediction outcome is due to the fact that cfRNA, originating from diverse tissues, contains substantial biological noise and lacks the specificity to accurately distinguish individuals at risk for AD from healthy control. Consequently, we proceeded to utilize brain single-cell RNA-sequencing (scRNA-seq) data to establish a direct link between cfRNA and genes associated with AD brain lesions.

### Non-invasive detection of cell-type-specific signatures in AD brains through blood cfRNA profiling

**FIGURE 2:**
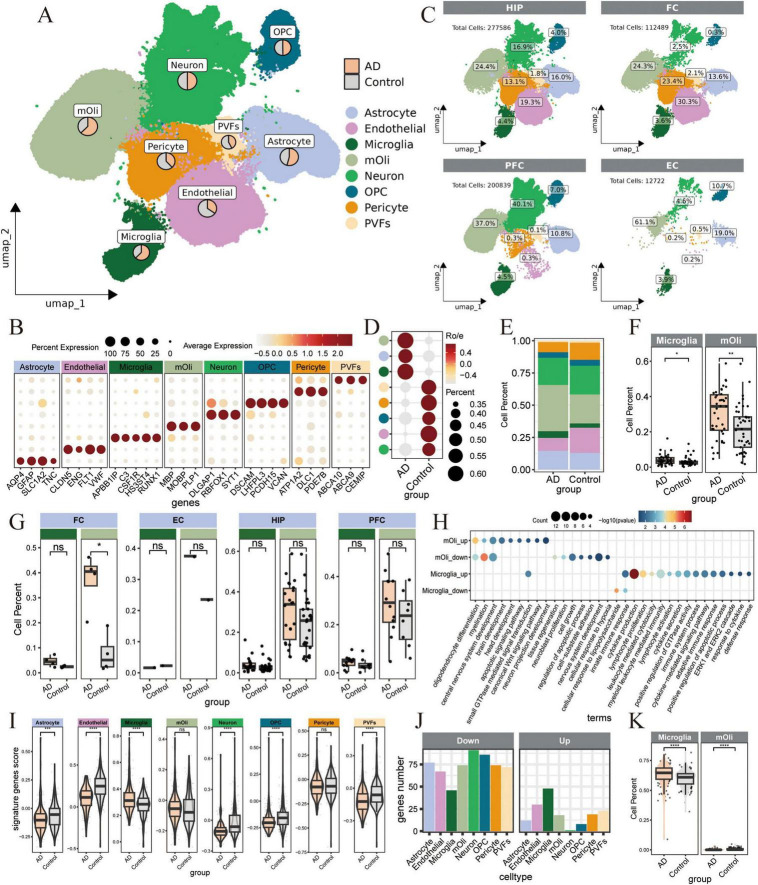
Unraveling cell-type-specific signatures in Alzheimer’s disease (AD) brains though non-invasive cell-free RNA (cfRNA) profiling. **(A)** Uniform Manifold Approximation and Projection (UMAP) plot shows the distribution of major cell types in brain tissues from AD and control patients, with cells colored by different cell types. The dataset includes a total of 603,636 single cells or nuclei. The pie chart shows the ratio of cells in AD and control groups. **(B)** Dot plot shows canonical marker genes in each cell type in panel **(A)**. Dot size indicates the proportion of expressing cells, colored by the average standardized expression levels. **(C)** Quantification of each cell types in the brain tissue at four different regions (HIP: hippocampus, FPC, frontal prefrontal cortex, FC, frontal cortex, EC, entorhinal cortex). **(D)** Dot plot shows the group preference for each cell type, measured by the ratio of observed to expected cell numbers (R_*o/e*_). The dot color represents the R_*o/e*_ value, while the dot size indicates the percentage of this cell population within the group. **(E)** Barplot shows the percentage of cells in AD and control samples from the single-cell data, with each bar colored according to the major cell types. **(F,G)** Box plot shows the proportions of Microglia and mOli in AD and control groups from single cells datasets (46 AD samples and 42 control samples). Similar analyses of cell proportion differences were also calculated in various brain regions. **(G)** *P*-values were calculated by the Wilcoxon rank-sum test. *for *p*_*adj*_ ≤ 0.05, **for *p*_*adj*_ ≤ 0.01, ***for *p*_*adj*_ ≤ 0.001, and ****for *p*_*adj*_ ≤ 0.0001. **(H)** Dot plot illustrates the representative Gene Ontology (GO) and Kyoto Encyclopedia of Genes and Genomes (KEGG) terms significantly (*p*_*adj*_ ≤ 0.05) enriched in up- and down-regulated DEGs in mOli and microgila cells, comparing AD samples with control samples. The dot color represents the - log10 *p*_*adj*_, while the dot size represents the number of genes associated with each terms. **(I)** Violin plot shows the score of the top 100 signature genes for each cell type in the cfRNA-seq dataset. The Wilcoxon rank-sum test was used to quantify the differences in score between the AD and control groups, with the following significance levels: *for *p*_*adj*_ ≤ 0.05, **for *p*_*adj*_ ≤ 0.01, ***for *p*_*adj*_ ≤ 0.001, and ****for *p*_*adj*_ ≤ 0.0001. **(J)** Barplot shows the number of upregulated or downregulated genes among the top 100 signature genes of each cell type in the cfRNA dataset, comparing 172 AD samples and 165 age-matched controls. **(K)** Box plot shows the Microglia and mOli cell proportions in AD and control groups by the BayesPrism deconvolution method in cfRNA dataset.

To investigate the molecular links between blood cfRNA and brain-specific changes in AD patients, we have integrated cfRNA data with scRNA-seq from AD brains tissues, and age-matched control. This integration includes scRNA-seq data from four distinct brain regions: hippocampus (HIP: GSE185553, GSE185277, GSE198323, GSE163577), frontal prefrontal cortex (FPC: GSE163577, GSE157827, GSE174367), frontal cortex (FC: GSE222494, GSE163577) and entorhinal cortex (EC: GSE138852). For detailed descriptions, refer to the Supplementary Table 1. After rigorous data quality control, integration, and batch correction steps, we obtained the single-cell and single-nucleus transcriptomes of 603,636 high-quality cells from 88 samples (46 AD patients and 42 control) (Supplementary Figures 2A–D) for further analysis. Eight major cell types in brains were annotated based on respective canonical marker genes. Visualization in Uniform Manifold Approximation and Projection (UMAP) space separated the clusters into astrocyte, endothelial, microglia, mature oligodendrocyte (mOli), neuron, oligodendrocyte precursor cell (OPC), pericyte, and perivascular fibroblasts (PVFs) (Figures 2A, B). All cell types were detected in the four brain regions: HIP, 277,586 cells; FC, 112,489 cells; PFC, 200,839 cells; and EC, 12,722 cells (Figure 2C). Microglia, mOli, and astrocyte cells showed comparable enrichment in AD patients (Figures 2D, E), consistent with findings from prior research (Lau et al., 2020; Yang et al., 2022). The activation of glial cells, particularly microglia and astrocytes, constitutes a significant pathological hallmark of AD and plays a key role in pathological states and participate in inflammatory responses (Deng et al., 2024; Kaur et al., 2019; Leng and Edison, 2021; Vandenbark et al., 2021). Specifically, this increase trend in the number of mOli and microglia cells was observed across various brain regions in AD (Figures 2F, G). The mOli population showed significant enrichment in the oligodendrocyte differentiation pathway, whereas microglia demonstrated upregulation of cytokine production, cytokine secretion, and inflammation-related pathways (Figure 2H). In contrast, PVFs, pericyte, and endothelial cells were more prevalent in aging control, while OPC and neuron remained relatively stable across both groups (Figures 2D, E).

Our analysis showed that the majority of signature genes (above 90%) of various brain cell types were detectable in cfRNA data (Supplementary Figure 3B), indicating that brain-derived RNAs can cross the blood-brain barrier and enter the bloodstream. Quantification of the expression levels of these signature genes in cfRNA data indicated up-regulation of microglia and mOli markers in AD (Figure 2I). Furthermore, the application of the BayesPrism deconvolution method (Chu et al., 2022) to estimate cell type proportions in cfRNA data revealed significant increases in microglia in AD (Figure 2K and Supplementary Figure 3E). In contrast, the signature genes expression scores for endothelial, neuron, OPC, pericyte, and PVFs cells were depleted in AD (Figure 2I), aligning with observed changes in cell proportions from single-cell data (Figures 2D, E). Reports indicate that astrocytes and microglia play a crucial role in regulating cell homeostasis, phagocytosis, and the clearance of Aβ and p-Tau, which significantly impacts the progression of AD. The observed downward trend in signature gene expression under AD conditions (Figure 2J) suggests a potential loss of normal cellular function, a phenomenon that warrants further investigation.

### Genetic concordance in blood and brain transcriptome linked to AD’s progression

**FIGURE 3:**
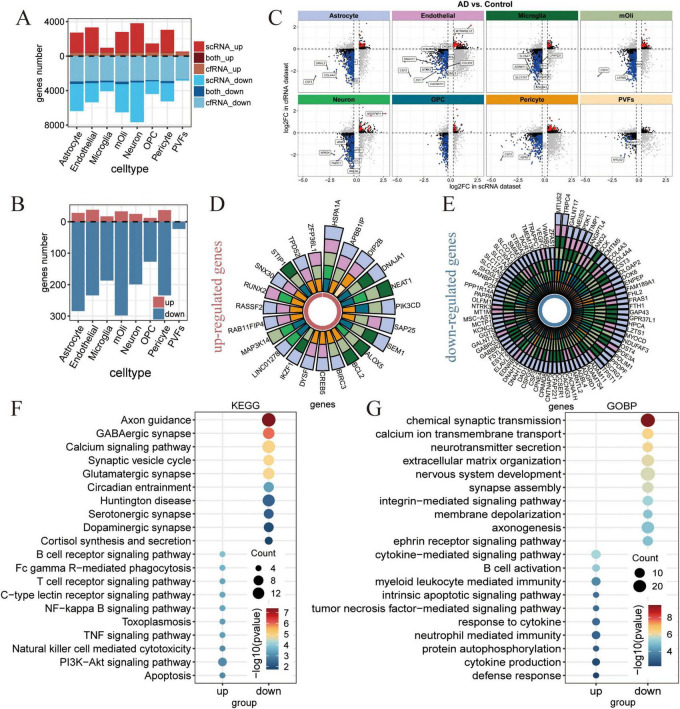
Identification of genes exhibiting consistent changes across blood and brain transcriptome data. **(A)** Barplot shows the number of up- regulated (red) and down-regulated (blue) differentially expressed genes (DEGs) in each cell types across single cell RNA sequencing (scRNA-seq) and cfRNA-seq datasets. **(B)** Barplot shows the number of overlapped up- and down-regulated DEGs in both scRNA-seq and cfRNA-seq datasets. **(C)** Scatter plots showing the log 2-fold change (log2FC) of intersecting DEGs between scRNA-seq and cfRNA-seq datasets. The x-axis represents the log2FC of DEGs from the scRNA-seq data, while the y-axis shows the log2FC of DEGs from the cfRNA-seq data, with each cell type displayed separately. Red indicates genes that are commonly up-regulated, blue indicates genes that are commonly down-regulated, and gray indicates genes with opposing trends. (*p*_*adj*_ ≤ 0.05 was used as the cutoff criteria for cfRNA-seq data; *p* ≤ 0.0_*adj*_5 and |log2FC| ≥ 0.25 was used as the cutoff criteria for scRNA-seq data). **(D)** Circle plot displays up-regulated DEGs shared by at least three cell types. The color key indicates different cell types, as in Figure 1A. **(E)** Circle plot displays down-regulated DEGs shared by at least three cell types. The color key indicates different cell types, as in Figure 1A. **(F,G)** Dot plots show the representative significantly Kyoto Encyclopedia of Genes and Genomes (KEGG) **(F)** and Gene Ontology (GO) **(G)** terms associated with the up-regulated and down-regulated DEGs from panel **(D,E)**.

While AD-associated transcriptomic alterations have been investigated in the brain regions (Grubman et al., 2019; Lau et al., 2020; Morabito et al., 2021; Yang et al., 2022; Zhou et al., 2022) or blood cfRNA data (Toden et al., 2020) separately, studies integrating single-cell transcriptomics from the brain with blood cfRNA transcriptomics to reveal consistent changes has yet to be reported. In this study, we aim to decipher and identify the consistent changes that indicate AD brain pathology, as detectable through non-invasive blood-based cfRNA data. To pinpoint the specific cell types underlying brain changes in AD within cfRNA, we conducted an integrated analysis of scRNA and cfRNA datasets. Due to the inherent sparsity of scRNA-seq data, we conducted differential expression analysis using the pseudo-bulk approach. We identified numerous genes with shared expression patterns in both cfRNA and scRNA datasets that exhibited a down-regulated trend in AD. Neuron, mOli, astrocyte, endothelial, microglia and pericyte were the primary contributors to dysregulated genes, whereas OPC and PVFs contributed the lowest (Figure 3A). Using scRNA-seq data, we identified genes that were dysregulated in AD brain tissue for each cell type, and a majority of these genes were also found to be dysregulated in blood cfRNA (Figures 3B, C and Supplementary Figure 3D). MTATP6P1, a mitochondrial ATP synthase pseudogene (Zhao et al., 2018), was up-regulated in both scRNA and cfRNA datasets and may influence cellular processes through regulatory mechanisms. SLC6A7, a member of the gamma-aminobutyric acid (GABA) neurotransmitter gene family, has been observed to be down-regulated, reflecting changes within the nervous system (Reid et al., 2022).

Interestingly, we discovered that a subset of genes exhibited dysregulated expression across multiple cell types in AD, and these genes were detectable in both blood cfRNA and brain tissues. For instance, BCL2, known for its role in inhibiting apoptosis in neurons (Eguchi et al., 1992; Yin et al., 1994), was found to be up-regulated in three cell types (Figure 3D). Conversely, MTUS2 (Figure 3E), which encodes a microtubule-associated scaffold protein playing a crucial role in late-onset Alzheimer’s disease (LOAD) (Xicota et al., 2024), exhibited decreased expression across seven distinct cell types. Overall, our analysis of the intersection of scRNA and cfRNA data revealed 23 up-regulated (Figure 3D) and 81 down-regulated (Figure 3E) genes. The down-regulated pathways, such as axon guidance, synaptic vesicle cycle, glutamatergic synapse, chemical synaptic transmission, neurotransmitter secretion, and nervous system development, are characteristic of neurodegenerative diseases, while the up-regulated pathways, closely associated with neuroinflammation, such as the T cell receptor signaling pathway, B cell differentiation, and cytokine-mediated signaling pathways, suggest an enhanced neuroinflammation in AD (Figures 3F, G). By integrating cfRNA and scRNA datasets, we offer a comprehensive depiction of AD’s progression in both the brain and blood, encompassing neuronal death and neuroinflammation, which are key pathological hallmarks of the disease.

### Establishment and verification of a multi-cfRNA-based classifier for AD diagnosis

The above results have indicated that cfRNA-based biomarkers lack the specificity necessary to accurately differentiate individuals at risk for AD from healthy control, resulting in an elevated false positive rate in AD diagnosis (Figure 1E). However, our research indicates that integrating cfRNA and scRNA data can more accurately reflect AD characteristics, offering a comprehensive view of the disease’s progression in both brain and blood. Consequently, we aim to develop AD classifiers by leveraging the intersection of biomarkers common to both cfRNA and scRNA datasets. This integrated approach is expected to capture a more comprehensive profile of AD, potentially enhancing the accuracy and reliability of our classifiers.

**FIGURE 4:**
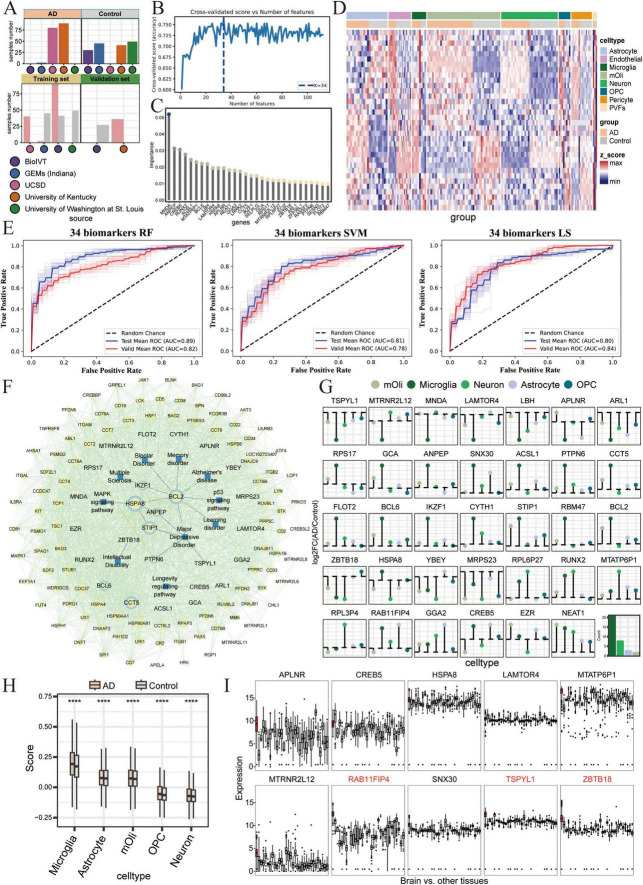
Performance of classifiers based on biomarker expression for Alzheimer’s disease (AD) diagnosis. **(A)** Schematics diagram illustrates the establishment of the classifiers in cfRNA-seq dataset. The training and independent validation sets included samples from different hospital sources. **(B)** Plot shows the cross-validated accuracy score versus the number of features, calculated by sklearn’s feature selection algorithms. **(C)** Lollipop plot shows the feature importance of top 34 representative biomarker genes by sklearn’s feature selection algorithms. **(D)** Heatmap shows the expression of 34 representative biomarker genes in single cell RNA-seq (scRNA-seq) dataset for each cell type. **(E)** The Receiver Operating Characteristic (ROC) curve and Area Under the Curve (AUC) value for the 34 biomarkers in the diagnosis of AD patients across three models in the cfRNA-seq datasets. Red represents the testing mean ROC curves, while blue represents the validation mean ROC curves. **(F)** The protein-protein interaction (PPI) network for the 34 biomarkers. Blue concentric circles represent the biomarkers, blue squares represent diseases, and yellow circles represent the intermediate genes. **(G)** The lollipop plot displays the log2FC values of the 34 biomarkers in each cell type from the scRNA-seq dataset. The color key indicates different cell types, as in Figure 1A. **(H)** Boxplot shows the score of the 34 biomarkers across AD and control groups, by cell types from scRNA-seq data. *P*- values were calulated by the Wilcoxon rank-sum test: **** for padj ≤ 0.0001. **(I)** Boxplots represent the expression of biomarkers genes across various tissues. Transcriptome data of tissues were obtained from the GTEx database. Red bars indicate the brain, while gray bars represent other tissues.

Upon conducting a coparative differential expression analysis between AD patients and age-matched control, we identified a total of 112 genes that were differentially expressed in both cfRNA and scRNA datasets (Supplementary Figure 4). To evaluate the generalizability of the models, the cfRNA cohort was partitioned into training (70%), test (20%), and validation (10%) sets for cross-validation (Figure 4A). Through feature selection with RFECV algorithm, 34 key biomarker genes were used to develop machine learning predictive models (Figures 4B, C). We established three distinct classifiers - SVM, RF, and LR. The RF model, which incorporated the cfRNA expression data of the 34 biomarker genes, achieved the highest AUC of 89% (Supplementary Figure 4). Both the SVM and LR classifiers also demonstrated high predictive performance (SVM: AUC = 0.81, LR: AUC = 0.80), and maintained this performance in the independent validation set (RF: AUC = 0.82, SVM: AUC = 0.78, LR: AUC = 0.84) (Figure 4E). These results indicates that combining single-cell transcriptomic data from the brain with blood-drived cfRNA analysis allows for a more precise capture of molecular alterations within the brain (Figure 4D). This integration enhances the predictive accuracy of cfRNA-based biomarkers in diagnosing AD.

To investigate the potential of our identified 34 biomarker genes to capture additional characteristics of AD, we performed the futher analysis on cfRNA and scRNA datasets, respectively. A total of 88 samples were clustered into AD patients and aging-matched healthy control based on these 34 genes expression profiling in scRNA-seq data (Figure 4D). An associative analysis with known disease databases identified BCL2 as the most significant hub gene, associated with a variety of diseases, such as bipolar disorder, memory disorder, learning disorder, AD, and major depressive disorder. Additionally, PTPN6, STIP1, ANPEP, and TSPYL1 were found to be correlated with major depressive disorder. Protein-Protein Interaction (PPI) analysis further highlighted BCL2, BCL6, HSPA8, and EZR as important hub genes (Figure 4F). Studies suggest that inhibiting BCL6 and BCL2 expression may serve as a therapeutic target for central nervous system cancer (Gourisankar et al., 2023). The HSPA8 gene functions as a molecular chaperone, mediating autophagy and affecting the hydrolysis of misfolded proteins (Stricher et al., 2013). Single-cell expression profiling revealed that the expression patterns of these 34 biomarker genes varied significantly across different cell types affected by AD. Most of the genes showed the greatest changes in microglia, followed by neuron, astrocyte, and oligodendrocyte (Figures 4G, H). Analysis of tissue-specific expression patterns from GTEx RNA-seq data revealed that the expression levels of several biomarker genes, including APLNR, MTATP6P1, MTRNR2L12, RAB11FIP4, SNX30, TSPYL1, and ZBTB18, were significantly higher in the brain compared to other tissues (Figure 4I). These findings raise the possibility that cfRNA profiles are influenced by underlying cell-type-specific transcriptional changes in the brain during AD progression, and may serve as an indirect window into disease-related pathological processes. While this aligns with potential pathological characterization, further validation is required to establish a direct causal link between cfRNA profiles and disease-specific mechanisms. Finally, we found that the 47 genes are associated with metabolic pathways such as mitochondria and ATP, as well as neurodegenerative diseases. In contrast, the 34 biomarker genes are more closely related to immunity (Supplementary Figures 3F, G). This comprehensive strategy paves the way for providing a novel perspective for early AD screening in clinical applications.

### Independent validation of the multi-cfRNA classifier in brain tissue RNA-seq cohorts

**FIGURE 5:**
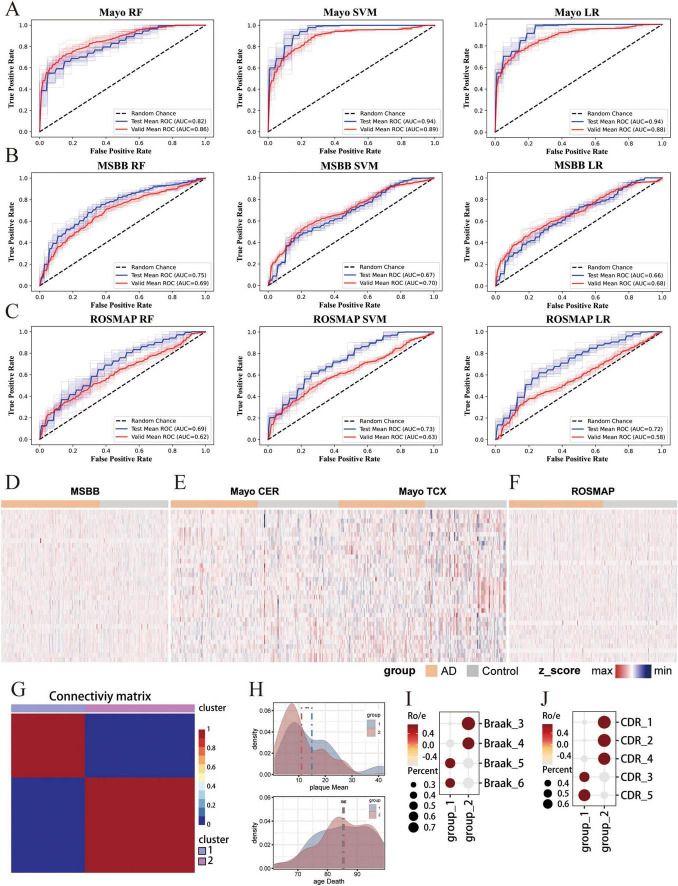
Independent validation of the multi-cfRNA classifier in brain tissue RNA-seq cohorts. **(A–C)** The Receiver Operating Characteristic (ROC) curves and Area Under the Curve (AUC) values for classifiers based on the expression of 34 biomarker genes in the diagnosis of AD patients in the Mayo **(A)** Religious Orders Study and Memory and Aging Project (ROSMAP) **(B)** and Mount Sinai Brain Bank (MSBB) **(C)** datasets. **(D–F)** Heatmaps showing the expression of 34 biomarker genes in Mayo/ROSMAP/MSBB data. **(G)** Heatmap shows unsupervised NMF clustering for MSBB AD samples based on the expression of 34 biomarkers. Two sample cluster subtypes are highlighted in color. **(H)** Density plot shows the plaque load values (top) and the age at death (bottom) in two AD groups AD samples. The Wilcoxon rank-sum test was then used to quantify the differences in scores, with the following significance levels: **for padj ≤ 0.01 and ns. for not significant. **(I)** Dot plot shows the Braaks preference for each NMF group, measured by the ratio of observed to expected cell numbers (R_o/e_). The dot color represents the R_o/e_ value, while the dot size indicates the percentage. **(J)** Dot plot shows the d Clinical Dementia Rating (CDR) score preference for each NMF group.

By integrating scRNA-seq data derived from the AD brain tissues, we successfully identified 34 key biomarker genes that can serve as biomarkers for screening individuals at risk of AD using blood-derived cfRNA-seq. To ascertain the applicability of these biomarker genes in constructing classifiers from brain tissue RNA-seq, we collected transcriptome data from three databases, ROSMAP, Mayo, and MSBB, which encompassed brain tissue samples from both AD patients and healthy control. The results showed that LR and SVM -based classifiers achieved the highest AUC of 94% in Mayo dataset based on the expression of 34 biomarker genes (Figure 5A). These genes also showed robust performance in the independent validation set, with AUC values consistently above 86% in three classifiers (Figure 5A). Moreover, these biomarker genes consistently achieved good predictive performance in both MSBB and ROSMAP datasets, demonstrating the predictive stability of the biomarker-based classifiers across different datasets (Figures 5B, C). In the training set, Mayo (RF: AUC = 0.82, SVM: AUC = 0.94, LR: AUC = 0. 94), MSBB (RF: AUC = 0.75, SVM: AUC = 0.67, LR: AUC = 0.66), ROSMAP (RF: AUC = 0.69, SVM: AUC = 0.73, LR: AUC = 0.72), and in the independent validation sets Mayo (RF: AUC = 0.86, SVM: AUC = 0.89, LR: AUC = 0.88), MSBB (RF: AUC = 0.69, SVM: AUC = 0.70, LR: AUC = 0.68), ROSMAP (RF: AUC = 0.62, SVM: AUC = 0.63, LR: AUC = 0.58) (Figures 5B, C). Consistently, no significant differences were observed in the expression levels of these 34 marker genes between the AD and control groups across the MSBB, Mayo, and ROSMAP datasets (Figures 5D–F).

Alzheimer’s disease is a slow and irreversible progressive neurodegenerative disease. Due to the lack of effective early detection methods, the disease is diagnosed at an advanced stage. Therefore, identifying the risk population at the early stages of is a major challenge in the field. To assess the potential of our identified biomarkers for early-stage screening, we have compiled RNA-seq data from brain tissues of AD patients spanning various disease stages.We applied Non-negative Matrix Factorization algorithm on the MSBB dataset to stratify AD samples into two distinct groups based on the expression of biomarker genes (Figure 5G). Group 1 is characterized by a higher mean plaque load (plaqueMean) and a shorter time to death (Figure 5H). Patients within this group exhibited elevated Braak stages and more pronounced Clinical Dementia Rating (CDR) scores, suggesting a more advanced stage of disease progression. Conversely, group 2 is marked by reduced mean plaque load, extended survival times, lower Braak stage and CDR scores. Patients within group 2 presented with mild symptoms and were classified as being in the early stage of AD (Figures 5I, J). Significantly, the 34 biomarker genes demonstrate a high capacity to distinguish early-stage AD patients. These results substantiate the utility of 34 biomarker genes in screening AD patients and underscore their potential for the early detection of AD.

## Discussion

In this study, we integrated high-throughput cfRNA-seq and scRNA-seq dataset from AD patients and age-matched control in multiple cohorts from blood or brain regions. Our results highlight the utility of integrating scRNA-seq data from brain tissues can better capture signatures from blood-derived cfRNA profiling that discriminate molecular variations in AD. Systematic profiling of cfRNA even non-invasively detect alterations in cell-type-specific signatures within the AD brain. We identified a total of 34 signature genes that were differentially expressed in both cfRNA and scRNA datasets. Machine learning algorithms (SVM, RF, and LR-based models) that utilize the cfRNA expression data from these 34 genes are capable of precisely distinguish patients with AD from healthy control (the highest AUC = 89%). Futhermore, classifiers developed based on the expression of 34 genes in brain transcriptome data also demonstrated robust predictive performance for assessing the risk of AD in the population (the highest AUC = 94%). The differential expression of 34 biomarker genes enable the identification of early-stage AD patients, which implying the potential application of these biomarkers in early AD screening, enabling the timely delivery of interventional treatments to high-risk individuals and potentially preventing disease progression. These results underscore the utility of the 34 identified genes as biomarkers for early, non-invasive AD screening, which could significantly improve diagnostic precision for AD.

Current treatments for AD, such as cholinesterase inhibitors, NMDA receptor antagonists (Liu et al., 2019), and therapies targeting Aβ and tau proteins (Congdon and Sigurdsson, 2018), primarily alleviate symptoms by modulating neurotransmitter levels. These treatments offer temporary relief or slow disease progression but do not stop disease advancement or provide a cure. To improve therapeutic outcomes, there is a need for biomarkers that can stratify AD patients and identify those likely to benefit from specific treatments. Therefore, non-invasive tools, more accurate than imaging or tissue biopsy, are needed to assess molecular profiles and drug response. Such tools could provide a foundation for clinical adjustments in treatment. Our findings show that cfRNA profiling can distinguish AD patients with different disease progressions, suggesting that the expression patterns of cfRNA reflect the heterogeneity of AD patients. CfRNA-based biomarkers may help develop indicators for drug response monitoring, supporting personalized treatment plans for AD patients.

Cell-free transcriptomes, characterized by their non-invasive nature, are emerging as valuable screening biomarkers for a variety of major diseases (Larson et al., 2021). Currently, exosomes and non-coding single-stranded RNAs (such as miRNAs) have demonstrated substantial promise in the field of cancer screening (Galvão-Lima et al., 2021; Kim and Croce, 2023), while their application in the diagnosis of neurodegenerative diseases remains in the exploratory phase. Additionally, the instability of RNA, which is prone to degradation, presents a major challenge in promoting the clinical use of cfRNA as biomarkers. Beyond transcriptomics, circulating cell-free DNA (cfDNA) and DNA methylation-based biomarkers also offer considerable clinical value. cfDNA is easily accessible and allows for repeated sampling, enabling real-time dynamic monitoring of disease status, making it particularly useful in applications like drug resistance testing. It exhibits high sensitivity in early cancer detection and is widely utilized in clinical settings (Bronkhorst et al., 2019) However, detecting cfDNA mutations often requires extremely deep sequencing, which introduces challenges such as false positives and increased costs, and is limited in scope and unable to trace tissue origins. DNA methylation-based biomarkers play a crucial role in chromatin transcription regulation, epigenetic gene expression, genomic stability, DNA repair, and replication, and are commercially available for testing (Levenson, 2010). Despite this, our AD diagnostic research has not yet incorporated these biomarkers due to a lack of blood-based cfDNA and methylation data. We intend to collect further data with the goal of identifying more precise and clinically relevant early AD screening biomarkers through integrated omics analyses.

In summary, we have demonstrated the capability of integrating single-cell transcriptomic data from the brain with cell-free transcriptomic data from blood, and their advantages over single-omics analyses in biomarker discovery. Brain data-derived biomarkers, while valuable, are not suitable for non-invasive clinical applications due to their invasive nature. Blood cell-free biomarkers suffer from high background noise that impedes the accurate reflection of brain pathologies. Therefore, the biomarkers identified in this study provide a significant resource, offering molecular insights into the pathogenesis of AD and facilitating the screening of early-stage AD patients. Ultimately, this strategy aims to achieve non-invasive early screening and precision medicine.

## Data Availability

The original contributions presented in this study are included in this article/Supplementary material, further inquiries can be directed to the corresponding authors.

## Footnotes

1
https://ggplot2.tidyverse.org

2
https://scikit-learn.org/1.0/

3
https://doi.org/10.7303/syn3219045

4
https://doi.org/10.7303/syn5550404

## Supplementary material

Supplementary Figure 1 Cell-free RNA-sequence (cfRNA-seq) data processing. **(A)** Barplot shows cfRNA-seq data sample information, with colors indicating different hospitals from which the cfRNA was sourced. **(B)** Principal component analysis (PCA) plot shows the batch effect present in the cfRNA-seq datasets. **(C)** PCA plot shows the batch correction by different hospitals. **(D)** Schematics diagram of cfRNA classifier establishment. The top side is the training set, and the bottom side is the independent validation set, separated according to the samples from different hospital sources. **(E)** Plot shows the cross-validated accuracy score versus the number of features, calculated using feature selection algorithms from sklearn. Ultimately, this process identified 47 biomarkers based on cfRNA-seq data. **(F)** Heatmap shows the expression levels of 47 biomarkers.

Supplementary Figure 2 Single-cell RNA-sequencing (scRNA-seq) data processing. **(A)** Barplot shows scRNA-seq data sample information, with colors indicating whether the samples are from the Alzheimer’s disease (AD) group or the control group. **(B)** Vlnplot shows the quality control information of scRNA-seq data, categorized according to the article sourced. **(C)** Uniform Manifold Approximation and Projection (UMAP) plot shows the batch effect originating from the article’s data sources. **(D)** The UMAP plot shows the results of batch correction applied to the data sourced from the article’s data sources. **(E)** Heatmap shows the Pearson correlation between cell types annotated in the scRNA-seq data with the following significance levels: *for *p*_*adj*_ ≤ 0.05, **for *p*_*adj*_ ≤ 0.01, ***for *p*_*adj*_ ≤ 0.001, and ****for *p*_*adj*_ ≤ 0.0001. **(F)** Barplot shows the cells number for each cell type, grouped by AD and control samples.

Supplementary Figure 3 Integrating single cell RNA-seq (scRNA-seq) and cfRNA-seq Data for biomarker identification and functional insights. **(A)** Scatter plots show the Spearman correlation between scRNA-seq data and cfRNA-seq data after log2 transformation of expression levels. **(B)** Barplot shows the detection rate of the scRNA-seq data top 100 signature genes in cfRNA-seq data, grouped by cell type. **(C)** The circle plot shows the up- and down-regulated genes that are shared between cfRNA-seq and scRNA-seq data. **(D)** Plot shows the up- and down-regulated genes number that are shared between cfRNA-seq and scRNA-seq data. **(E)** Barplot shows the cell proportions across Alzheimer’s disease (AD) and control groups, as determined by the BayesPrism deconvolution method in cfRNA data, with each bar representing a sample. **(F)** Barplot shows the representative significantly (*p*_*adj*_ ≤ 0.05) enriched Gene Ontology (GO) and Kyoto Encyclopedia of Genes and Genomes (KEGG) terms associated with 47 biomarkers. **(G)** Barplot shows the representative significantly enriched GO terms associated with 34 biomarkers.

Supplementary Figure 4 Schematic workflow for feature selection, model training, and independent validation.
