# Supplementary figures and images for: Integrative single-cell and cell-free plasma RNA transcriptomics identifies biomarkers for early non-invasive AD screening
Source: Front Aging Neurosci. 2025 May 30;17:1571783. doi: 10.3389/fnagi.2025.1571783 (PMC12162594; doi:10.3389/fnagi.2025.1571783)

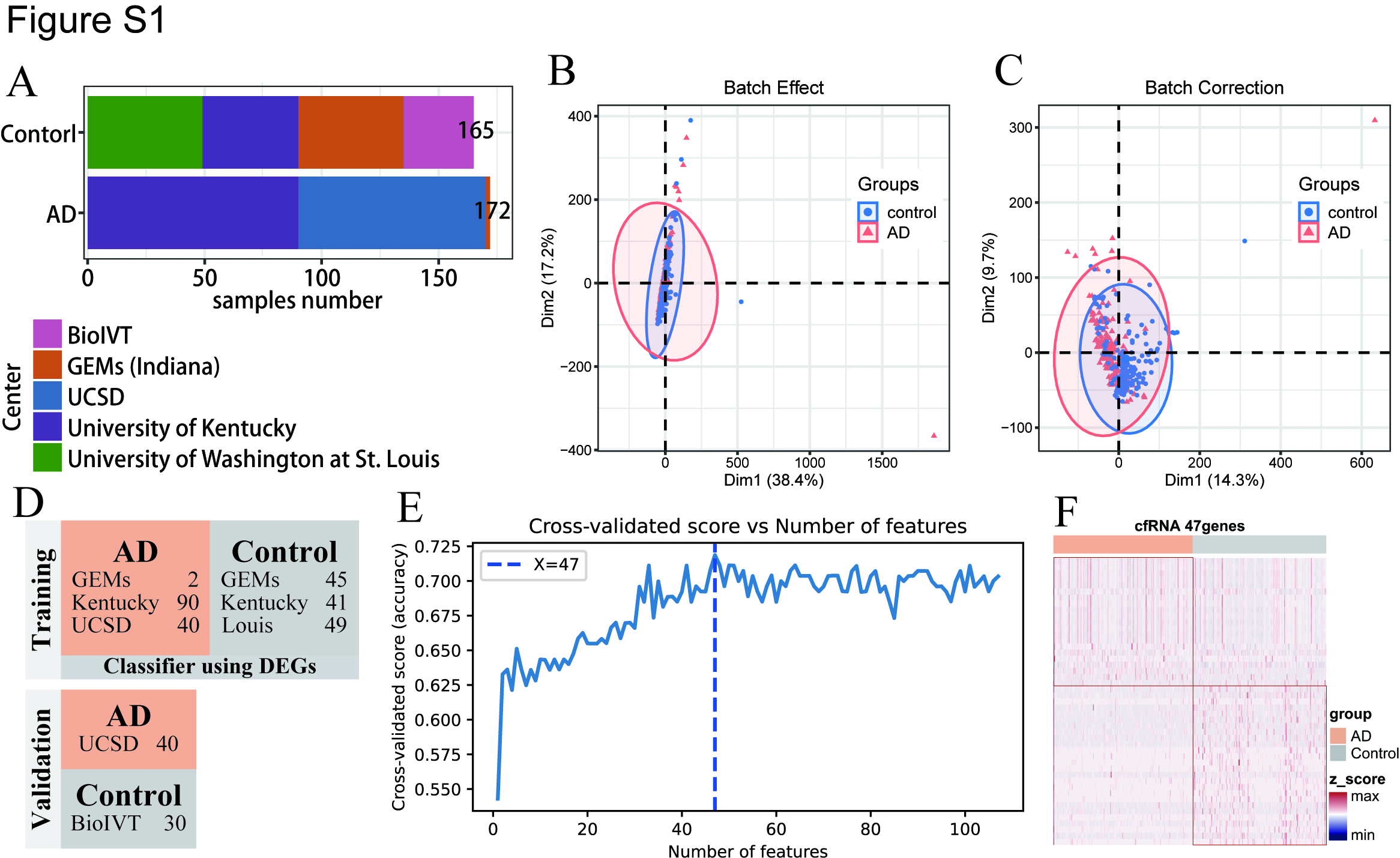

Supplement: Supplementary Figure 1 — Cell-free RNA-sequence (cfRNA-seq) data processing. (A) Barplot shows cfRNA-seq data sample information, with colors indicating different hospitals from which the cfRNA was sourced. (B) Principal component analysis (PCA) plot shows the batch effect present in the cfRNA-seq datasets. (C) PCA plot shows the batch correction by different hospitals. (D) Schematics diagram of cfRNA classifier establishment. The top side is the training set, and the bottom side is the independent validation set, separated according to the samples from different hospital sources. (E) Plot shows the cross-validated accuracy score versus the number of features, calculated using feature selection algorithms from sklearn. Ultimately, this process identified 47 biomarkers based on cfRNA-seq data. (F) Heatmap shows the expression levels of 47 biomarkers. [file Image_1.tif]

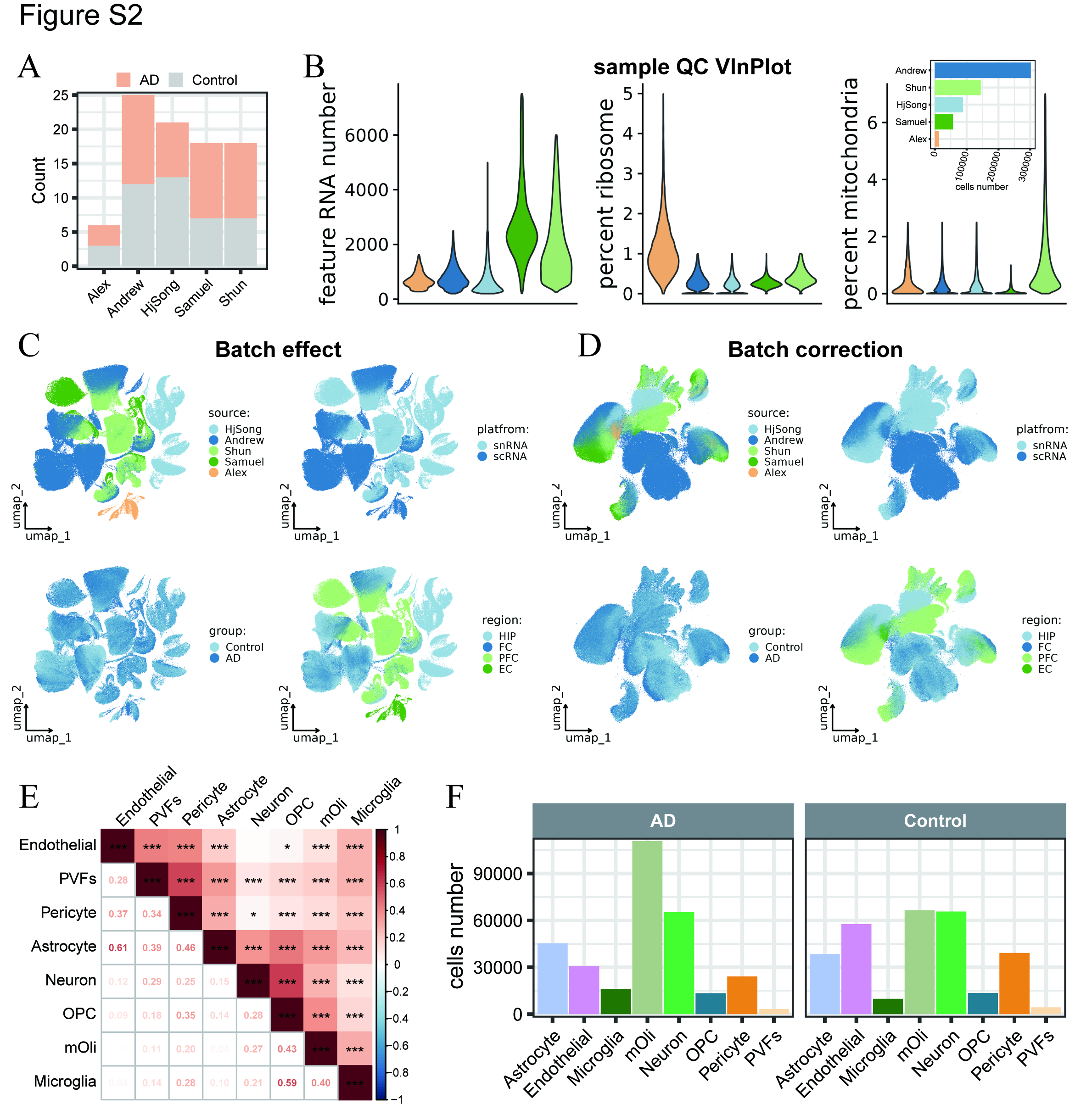

Supplement: Supplementary Figure 2 — Single-cell RNA-sequencing (scRNA-seq) data processing. (A) Barplot shows scRNA-seq data sample information, with colors indicating whether the samples are from the Alzheimer’s disease (AD) group or the control group. (B) Vlnplot shows the quality control information of scRNA-seq data, categorized according to the article sourced. (C) Uniform Manifold Approximation and Projection (UMAP) plot shows the batch effect originating from the article’s data sources. (D) The UMAP plot shows the results of batch correction applied to the data sourced from the article’s data sources. (E) Heatmap shows the Pearson correlation between cell types annotated in the scRNA-seq data with the following significance levels: *for padj ≤ 0.05, **for padj ≤ 0.01, ***for padj ≤ 0.001, and ****for padj ≤ 0.0001. (F) Barplot shows the cells number for each cell type, grouped by AD and control samples. [file Image_2.tif]

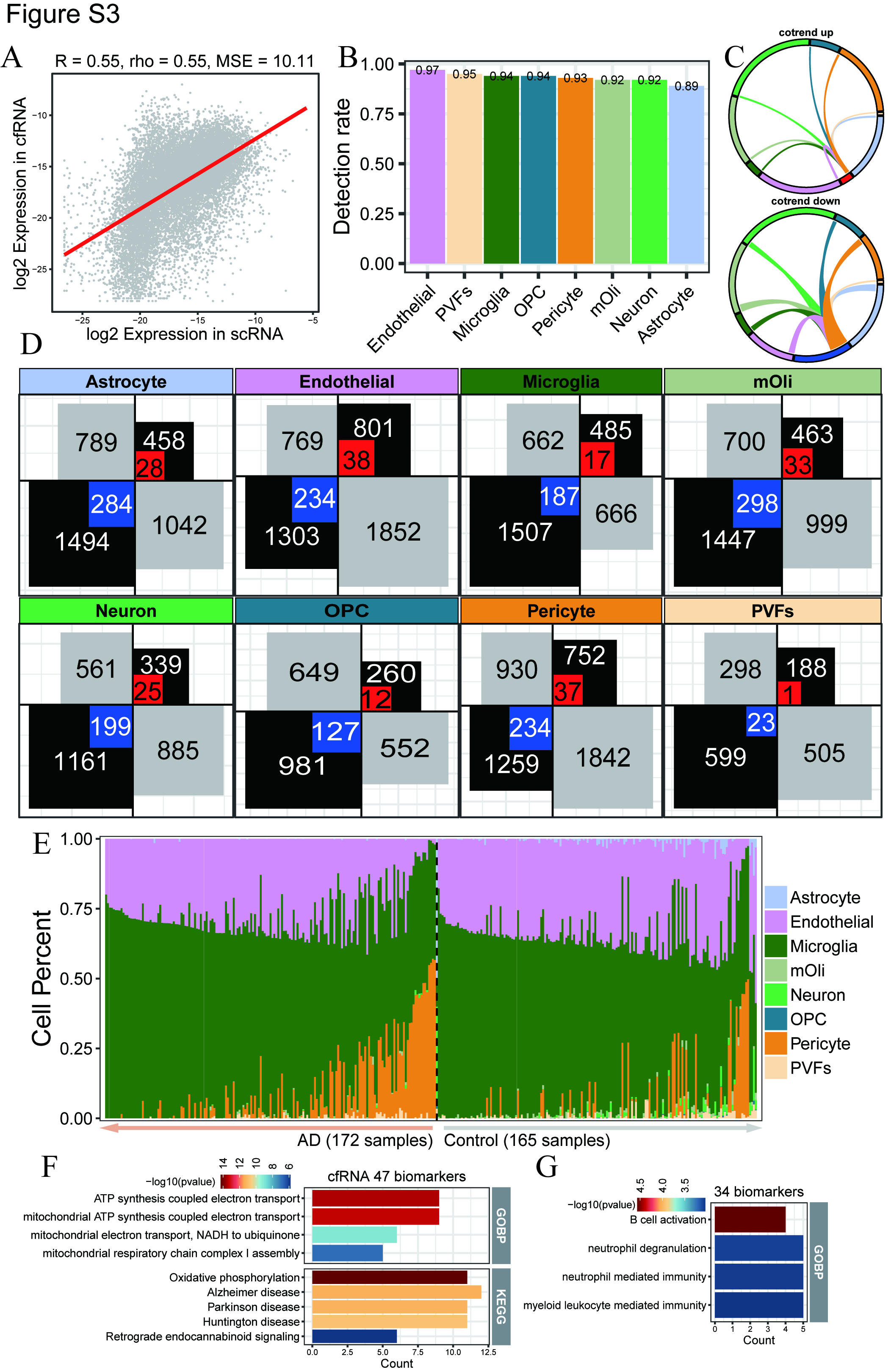

Supplement: Supplementary Figure 3 — Integrating single cell RNA-seq (scRNA-seq) and cfRNA-seq Data for biomarker identification and functional insights. (A) Scatter plots show the Spearman correlation between scRNA-seq data and cfRNA-seq data after log2 transformation of expression levels. (B) Barplot shows the detection rate of the scRNA-seq data top 100 signature genes in cfRNA-seq data, grouped by cell type. (C) The circle plot shows the up- and down-regulated genes that are shared between cfRNA-seq and scRNA-seq data. (D) Plot shows the up- and down-regulated genes number that are shared between cfRNA-seq and scRNA-seq data. (E) Barplot shows the cell proportions across Alzheimer’s disease (AD) and control groups, as determined by the BayesPrism deconvolution method in cfRNA data, with each bar representing a sample. (F) Barplot shows the representative significantly (padj ≤ 0.05) enriched Gene Ontology (GO) and Kyoto Encyclopedia of Genes and Genomes (KEGG) terms associated with 47 biomarkers. (G) Barplot shows the representative significantly enriched GO terms associated with 34 biomarkers. [file Image_3.tif]

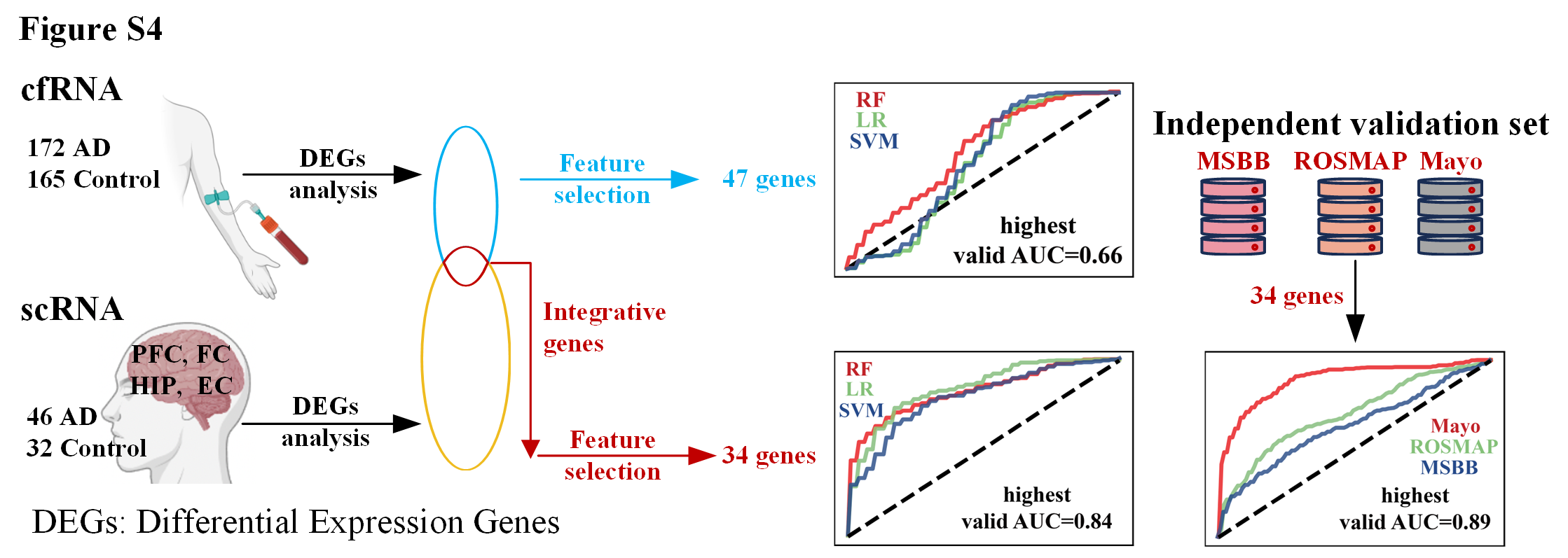

Supplement: Supplementary Figure 4 — Schematic workflow for feature selection, model training, and independent validation. [file Image_4.tif]
